# Supplementary material for: A Multi-Matrix Metabolomic Approach in Ringed Seals and Beluga Whales to Evaluate Contaminant and Climate-Related Stressors
Source: Metabolites. 2022 Aug 30;12(9):813. doi: 10.3390/metabo12090813 (PMC9502077; doi:10.3390/metabo12090813)
Supplement: Supplementary file 1 [file metabolites-12-00813-s001.zip › metabolites-1729985-supplementary.pdf]

metabolites

MDPI

Article

A Multi-Matrix Metabolomic Approach in Ringed Seals and Beluga Whales to Evaluate Contaminant and Climate-Related Stressors

Antoine É. Simond [1,2], Marie Noël [3], Lisa Loseto [4,5], Magali Houde [6], Jane Kirk [7], Ashley Elliott [4] and Tanya M. Brown [1,2,*]

1 Pacific Science Enterprise Centre, Fisheries and Oceans Canada, 4160 Marine Drive, West Vancouver, BC V7V 1N6, Canada
2 School of Resource and Environmental Management, Simon Fraser University, 4160 Marine Drive, West Vancouver, BC V7V 1N6, Canada
3 Ocean Wise, 101-440 Cambie Street, Vancouver, BC V6B 2N5, Canada
4 Freshwater Institute, Fisheries and Oceans Canada, 501 University Crescent, Winnipeg, MB R3T 2N6, Canada
5 Centre for Earth Observation Science, University of Manitoba, Winnipeg, MB R3T 2N2, Canada
6 Centre St-Laurent, Environment and Climate Change Canada, 105 McGill Street, Montreal, QC H2Y 2E7, Canada
7 Canada Centre for Inland Waters, Environment and Climate Change Canada, 867 Lakeshore Road, Burlington, ON L7S 1A1, Canada
* Correspondence: tanya.brown@dfo-mpo.gc.ca

metabolites MDPI

Supplementary Materials

Table S1. Metabolite classes, number of metabolites quantified per class (*n*) and geometric mean (± standard deviation) concentrations (µg/g) determined in each tissue of male ringed seals (*n* = 9) collected in 2019 in Lake Melville, Labrador, Canada.

**Metabolite classes**	**Plasma**		**Liver**		**Inner blubber**		**Outer blubber**	
	n	**[ ]**	***n***	**[ ]**	***n***	**[ ]**	***n***	**[ ]**
Acylcarnitines	11	1.98 ± 0.56	29	125 ± 46.3	6	2.75 ± 1.63	6	3.12 ± 0.90
Amino acids	20	486 ± 145	21	2,122 ± 319	20	101 ± 51.7	20	149 ± 43.4
Bile acids	4	0.20 ± 0.68	4	49.0 ± 30.4	0	-	0	-
Biogenic amines	15	53.5 ± 15.6	11	140 ± 131	10	103 ± 47.1	10	120 ± 20.0
Carbohydrates	1	1,186 ± 249	1	2,552 ± 1,615	1	27.3 ± 33.5	1	44.9 ± 60.6
Energy metabolites	6	934 ± 344	12	4,024 ± 1,030	6	168 ± 128	6	199 ± 101
Fatty acids	10	173 ± 120	6	228 ± 100	6	308 ± 562	6	119 ± 153
Lysophosphatidylcholines	11	150 ± 32.1	9	163 ± 101	4	9.21 ± 4.57	4	8.81 ± 2.56
Phosphatidylcholines	75	530 ± 143	75	2,054 ± 305	68	526 ± 139	68	582 ± 52.4
Sphingolipids	14	413 ± 56.2	14	1,227 ± 85.2	13	193 ± 59.1	13	226 ± 51.9
**Total**	**167**	**4,017 ± 721**	**182**	**13,105 ± 2,652**	**134**	**1,588 ± 802**	**134**	**1,575 ± 186**

Table S2. Metabolite classes, number of metabolites quantified per class (*n*) and geometric mean (± standard deviation) concentrations (μg/g) determined in each tissue of male Eastern Beaufort Sea belugas, Northwest Territories, Canada, collected in 2009 (*n* = 4) and 2017 (*n* = 9).

**Metabolite classes**	**Plasma** [a]		**Liver** [b]		**Inner blubber** [c]		**Outer blubber** [c]	
	n	**[ ]**	***n***	**[ ]**	***n***	**[ ]**	***n***	**[ ]**
Acylcarnitines	16	2.05 ± 1.23	17	26.3 ± 1.61	10	11.0 ± 6.78	10	14.8 ± 6.48
Amino acids	18	632 ± 123	21	3,664 ± 641	21	269 ± 642	21	264 ± 509
Bile acids	3	0.25 ± 0.42	2	37.3 ± 22.4	0	-	0	-
Biogenic amines	16	98.3 ± 38.3	16	1,643 ± 133	13	193 ± 186	13	199 ± 163
Carbohydrates	1	1,100 ± 616	1	13,345 ± 1,607	1	254 ± 795	1	228 ± 1,127
Energy metabolites	6	105 ± 67.3	11	4,515 ± 355	4	468 ± 898	4	416 ± 764
Fatty acids	7	15.3 ± 35.8	11	4,307 ± 815	8	399 ± 458	8	216 ± 391
Lysophosphatidylcholines	9	77.0 ± 41.5	12	438 ± 33.8	5	12.9 ± 13.9	5	8.49 ± 9.94
Phosphatidylcholines	73	324 ± 96.3	75	3,101 ± 280	72	562 ± 259	72	595 ± 309
Sphingomyelins	14	166 ± 70.6	14	1,327 ± 146	12	236 ± 165	12	232 ± 162
**Total**	**163**	**2,657 ± 832**	**180**	**32,525 ± 1,261**	**146**	**2,963 ± 2,647**	**146**	**2,509 ± 2,805**

[a] Mean and SD of the belugas sampled in 2017 (*n* = 9) and 2009 (*n* = 2 instead of 4 as 2009-HI-09 and 2009-HI-10 belugas were outliers).
[b] Mean and SD of the belugas sampled in 2009 (*n* = 4).
[c] Mean and SD of the belugas sampled in 2017 (*n* = 9).

Table S3. Correlations between explanatory variables and metabolites that were correlated with a dimension of PCAs made for each tissue (see Figure 3) in ringed seals from Lake Melville. Correlations presented in this table were significant but did not pass the FDR adjustment, and thus were not considered significant based on our criteria. The full name of each metabolite can be found in Table S5.

Explanatory variable	Class	Metabolite	Tissue	Correlation coefficient	*p*-value
Muscle $\delta^{13}C$	Phosphatidylcholine	PC aa C32:3	Plasma	-0.67	0.050
	Phosphatidylcholine	PC aa C36:1	Plasma	-0.75	0.019
	Phosphatidylcholine	PC aa C38:1	Plasma	-0.79	0.011
	Phosphatidylcholine	PC aa C38:6	Plasma	-0.74	0.022
	Phosphatidylcholine	PC aa C40:1	Plasma	-0.79	0.012
	Phosphatidylcholine	PC aa C40:2	Plasma	-0.78	0.012
	Phosphatidylcholine	PC aa C42:2	Plasma	-0.72	0.028
	Phosphatidylcholine	PC aa C42:5	Plasma	-0.74	0.023
	Phosphatidylcholine	PC aa C42:6	Plasma	-0.69	0.040
	Phosphatidylcholine	PC aa C48:0	Plasma	-0.69	0.040
	Phosphatidylcholine	PC ae C34:1	Plasma	-0.71	0.031
	Phosphatidylcholine	PC ae C36:3	Plasma	-0.77	0.014
	Phosphatidylcholine	PC ae C38:2	Plasma	-0.69	0.042
	Phosphatidylcholine	PC ae C40:2	Plasma	-0.76	0.017
Girth	Phosphatidylcholine	PC ae C36:5	Liver	-0.85	0.004
	Sphingomyelin	SM C22:3	Liver	-0.94	< 0.001
HBB concentration	Fatty acid	FA C22:5n3c1	Inner blubber	0.68	0.045
	Fatty acid	FA C22:5n3c2	Inner blubber	0.68	0.043
	Phosphatidylcholine	PC aa C34:3	Inner blubber	-0.64	0.046
	Phosphatidylcholine	PC ae C34:0	Inner blubber	-0.71	0.033
	Sphingomyelin	SM C18:1	Inner blubber	-0.68	0.044
	Acylcarnitine	AC C5	Outer blubber	0.67	0.047
	Biogenic amine	Carnosine	Outer blubber	0.81*	0.008
	Carbohydrate	Hex	Outer blubber	-0.70	0.036
	Lysophosphatidylcholine	lysoPC a C18:1	Outer blubber	-0.75*	0.019
	Phosphatidylcholine	PC aa C34:1	Outer blubber	-0.83	0.006
	Phosphatidylcholine	PC aa C36:3	Outer blubber	-0.80	0.010
	Phosphatidylcholine	PC ae C34:1	Outer blubber	-0.67	0.047
	Phosphatidylcholine	PC ae C34:3	Outer blubber	-0.69	0.040
	Phosphatidylcholine	PC ae C36:3	Outer blubber	-0.84	0.005
Hg concentration	Phosphatidylcholine	PC ae C36:5	Liver	-0.85	0.004
	Sphingomyelin	SM C22:3	Liver	-0.76	0.017

* Correlation was evaluated using the Spearman coefficient as the metabolite percent contribution was not normal.

Table S4. Correlations between explanatory variables and metabolites that were correlated with a dimension of PCAs made for each tissue (see Figure 3) in Eastern Beaufort Sea belugas. Correlations presented in this table were significant but did not pass the FDR adjustment, and thus were not considered significant based on our criteria. The full name of each metabolite can be found in Table S5.

Explanatory variable	Class	Metabolite	Tissue	Correlation coefficient	*p*-value
Liver $\delta^{13}C$	Acylcarnitine	AC C12	Plasma	0.73	0.011
	Amino acid	Asn	Plasma	-0.65	0.029
	Fatty acid	FA C20:5	Plasma	0.75	0.008
	Fatty acid	FA C22:5n3c1	Plasma	0.64	0.033
	Fatty acid	FA C22:5n3c2	Plasma	0.76	0.007
	Fatty acid	FA C22:5n6c	Plasma	0.70	0.017
	Fatty acid	FA C22:6	Plasma	0.81	0.002
	Biogenic amine	Met SO	Plasma	-0.69	0.020
	Phosphatidylcholine	PC aa C30:0	Plasma	0.64	0.034
	Phosphatidylcholine	PC aa C30:2	Plasma	-0.81	0.002
	Phosphatidylcholine	PC aa C32:1	Plasma	-0.78	0.005
	Phosphatidylcholine	PC aa C32:2	Plasma	-0.85	0.001
	Phosphatidylcholine	PC aa C32:3	Plasma	-0.70	0.017
	Phosphatidylcholine	PC aa C34:1	Plasma	-0.65	0.032
	Phosphatidylcholine	PC aa C34:2	Plasma	-0.77	0.005
	Phosphatidylcholine	PC aa C34:3	Plasma	-0.79	0.004
	Phosphatidylcholine	PC aa C36:1	Plasma	-0.62	0.040
	Phosphatidylcholine	PC aa C36:2	Plasma	-0.70	0.016
	Phosphatidylcholine	PC aa C36:3	Plasma	-0.66	0.026
	Phosphatidylcholine	PC aa C36:6	Plasma	-0.65	0.031
	Phosphatidylcholine	PC aa C38:3	Plasma	-0.63	0.039
	Phosphatidylcholine	PC ae C32:1	Plasma	-0.68	0.021
	Phosphatidylcholine	PC ae C34:1	Plasma	-0.69	0.019
	Phosphatidylcholine	PC ae C34:3	Plasma	-0.70	0.016
	Phosphatidylcholine	PC ae C36:5	Plasma	-0.70	0.016
	Phosphatidylcholine	PC ae C38:5	Plasma	-0.65	0.029
	Phosphatidylcholine	PC ae C44:3	Plasma	-0.63	0.037
	Biogenic amine	Putrescine	Plasma	-0.64	0.034
	Sphingomyelin	SM C18:0	Plasma	0.63	0.040
	Phosphatidylcholine	PC aa C34:4	Inner blubber	-0.67	0.049
	Sphingomyelin	SM C24:1	Inner blubber	-0.76	0.017
Girth	Amino acid	Asp	Plasma	-0.65	0.031
Hg concentration	Acylcarnitine	AC C18:1	Outer blubber	-0.70	0.037
	Amino acid	Asp	Outer blubber	-0.70	0.035
	Biogenic amine	Carnosine	Outer blubber	-0.72	0.030
	Amino acid	Met	Outer blubber	-0.67	0.049
	Phosphatidylcholine	PC aa C36:6	Outer blubber	-0.77	0.014
	Phosphatidylcholine	PC aa C38:5	Outer blubber	-0.68	0.043
	Phosphatidylcholine	PC aa C38:6	Outer blubber	-0.78	0.014
	Phosphatidylcholine	PC aa C40:2	Outer blubber	-0.72	0.028

	Phosphatidylcholine	PC aa C40:5	Outer blubber	-0.77	0.015
	Phosphatidylcholine	PC aa C40:6	Outer blubber	-0.76	0.018
	Phosphatidylcholine	PC aa C42:2	Outer blubber	-0.67	0.049
	Phosphatidylcholine	PC aa C42:6	Outer blubber	-0.85	0.004
	Phosphatidylcholine	PC ae C34:0	Outer blubber	-0.89	0.001
	Phosphatidylcholine	PC ae C36:0	Outer blubber	-0.77	0.014
	Phosphatidylcholine	PC ae C36:5	Outer blubber	-0.68	0.045
	Phosphatidylcholine	PC ae C38:0	Outer blubber	-0.77	0.015
	Phosphatidylcholine	PC ae C38:2	Outer blubber	-0.67	0.049
	Phosphatidylcholine	PC ae C38:5	Outer blubber	-0.74	0.021
	Phosphatidylcholine	PC ae C38:6	Outer blubber	-0.70	0.037
	Phosphatidylcholine	PC ae C40:5	Outer blubber	-0.73	0.025
	Phosphatidylcholine	PC ae C40:6	Outer blubber	-0.69	0.039
	Phosphatidylcholine	PC ae C42:5	Outer blubber	-0.68	0.042
	Sphingomyelin	SM (OH) C14:1	Outer blubber	-0.79	0.012
	Sphingomyelin	SM C16:0	Outer blubber	-0.83	0.006
	Sphingomyelin	SM C16:1	Outer blubber	-0.68	0.046
	Sphingomyelin	SM C18:1	Outer blubber	-0.68	0.046
	Amino acid	Thr	Outer blubber	-0.77	0.016
Length	Acylcarnitine	AC C14:1	Inner blubber	0.83	0.006
	Acylcarnitine	AC C18:2	Inner blubber	0.68	0.043
	Phosphatidylcholine	PC aa C36:0	Inner blubber	0.70	0.034
PBDE concentration	Biogenic amine	GABA	Plasma	0.75*	0.010
	Amino acid	Glu	Plasma	0.70	0.016
	Lysophosphatidylcholine	lysoPC a C16:1	Plasma	-0.66	0.028
	Amino acid	Ala	Outer blubber	-0.74	0.023
	Fatty acid	FA C20:2	Outer blubber	0.76	0.018
	Amino acid	Gln	Outer blubber	-0.67	0.047
	Phosphatidylcholine	PC aa C38:0	Outer blubber	-0.79	0.011

* Correlation was evaluated using the Spearman coefficient as the metabolite percent contribution was not normal.

Table S5. List of metabolites analyzed in plasma, liver and blubber of ringed seal and beluga samples, with corresponding abbreviations and identification numbers from the human metabolome database (HMDB).

Target	Abbreviation	HMDB ID
Acylcarnitines		
D-Carnitine	AC C0	HMDB0000062
Acetylcarnitine	AC C2	HMDB0000201
Propionylcarnitine	AC C3	HMDB0000824
Hydroxypropionylcarnitine	AC C3-OH	HMDB0013125
Propenoylcarnitine	AC C3:1	HMDB0013124
Butyrylcarnitine	AC C4	HMDB0002013
Hydroxybutyrylcarnitine	AC C4-OH (C3-DC)	HMDB0013127
Butenylcarnitine	AC C4:1	HMDB0013126
Valerylcarnitine	AC C5	HMDB0013128
Glutarylcarnitine (Hydroxyhexanoylcarnitine)	AC C5-DC (C6-OH)	HMDB0061633
Methylglutarylcarnitine	AC C5-M-DC	HMDB0000552
Hydroxyvalerylcarnitine (Methylmalonylcarnitine)	AC C5-OH (C3-DC-M)	HMDB0013133
Tiglylcarnitine	AC C5:1	HMDB0002366
Glutaconylcarnitine	AC C5:1-DC	HMDB0013129
Hexanoylcarnitine (Fumarylcarnitine)	AC C6 (C4:1-DC)	HMDB0000756
Hexenoylcarnitine	AC C6:1	HMDB0013161
Pimelylcarnitine	AC C7-DC	HMDB0013328
Octanoylcarnitine	AC C8	HMDB0000791
Nonaylcarnitine	AC C9	HMDB0013288
Decanoylcarnitine	AC C10	HMDB0000651
Decenoylcarnitine	AC C10:1	HMDB0013205
Decadienylcarnitine	AC C10:2	HMDB0006469
Dodecanoylcarnitine	AC C12	HMDB0002250
Dodecanedioylcarnitine	AC C12-DC	HMDB0013327
Dodecenoylcarnitine	AC C12:1	HMDB0013326
Tetradecanoylcarnitine	AC C14	HMDB0005066
Tetradecenoylcarnitine	AC C14:1	HMDB0013329
Hydroxytetradecenoylcarnitine	AC C14:1-OH	HMDB0013330
Tetradecadienylcarnitine	AC C14:2	HMDB0013331
Hydroxytetradecadienylcarnitine	AC C14:2-OH	HMDB0013332
Hexadecanoylcarnitine	AC C16	HMDB0000222
Hydroxyhexadecanoylcarnitine	AC C16-OH	HMDB0013336
Hexadecenoylcarnitine	AC C16:1	HMDB0013207
Hydroxyhexadecenoylcarnitine	AC C16:1-OH	HMDB0013333
Hexadecadienylcarnitine	AC C16:2	HMDB0013334
Hydroxyhexadecadienylcarnitine	AC C16:2-OH	HMDB0013335
Stearoylcarnitine	AC C18	HMDB0000848
Octadecenoylcarnitine	AC C18:1	HMDB0094687
Hydroxyoctadecenoylcarnitine	AC C18:1-OH	HMDB0013339
Octadecadienylcarnitine	AC C18:2	HMDB0006469
Amino acids		
Alanine	Ala	HMDB0000161
Arginine	Arg	HMDB0000517
Asparagine	Asn	HMDB0000168
Aspartic acid	Asp	HMDB0000191

Citrulline	Cit	HMDB0000904
Glutamine	Gln	HMDB0000641
Glutamic acid	Glu	HMDB0000148
Glycine	Gly	HMDB0000123
Histidine	His	HMDB0000177
Isoleucine	Ile	HMDB0000172
Leucine	Leu	HMDB0000687
Lysine	Lys	HMDB0000182
Methionine	Met	HMDB0000696
Ornithine	Orn	HMDB0000214
Phenylalanine	Phe	HMDB0000159
Proline	Pro	HMDB0000162
Serine	Ser	HMDB0000187
Threonine	Thr	HMDB0000167
Tryptophan	Trp	HMDB0000929
Tyrosine	Tyr	HMDB0000158
Valine	Val	HMDB0000883
Bile acids		
Cholic acid	CA	HMDB0000619
Chenodeoxycholic acid	CDCA	HMDB0000518
Deoxycholic acid	DCA	HMDB0000626
Glycocholic acid	GCA	HMDB0000138
Glycochenodeoxycholic acid	GCDCA	HMDB0000637
Glycodeoxycholic acid	GDCA	HMDB0000631
Lithocholic acid	LCA	HMDB0000761
Taurocholic acid	TCA	HMDB0000036
Taurochenodeoxycholic acid	TCDCA	HMDB0000951
Taurodeoxycholic acid	TDCA	HMDB0000896
Taurolithocholic acid	TLCA	HMDB0000722
Tauroursodeoxycholic acid	TUDCA	HMDB0000874
Ursodeoxycholic acid	UDCA	HMDB0000946
Biogenic amines		
Acetyl-ornithine	Ac-Orn	HMDB0003357
Asymmetric dimethylarginine	ADMA	HMDB0001539
α-aminoadipic acid	α-AAA	HMDB0000510
Carnosine	Carnosine	HMDB0000033
Creatinine	Creatinine	HMDB0000562
Dopa	DOPA	HMDB0000181
Dopamine	Dopamine	HMDB0000073
gamma-Aminobutyric acid	GABA	HMDB0000112
Histamine	Histamine	HMDB0000870
Hydroxyproline	Hyp	HMDB0000725
Kynurenine	Kynurenine	HMDB0000684
Methionine sulfoxide	Met-SO	HMDB0002005
Nitrotyrosine	Nitro-Tyr	HMDB0001904
Phenylethylamine	PEA	HMDB0012275
Putrescine	Putrescine	HMDB0001414
Sarcosine	Sarcosine	HMDB0000271
Symmetric dimethylarginine	SDMA	HMDB0003334
Serotonin	Serotonin	HMDB0000259

Spermidine	Spermidine	HMDB0001257
Spermine	Spermine	HMDB0001256
Taurine	Taurine	HMDB0000251
Carbohydrates		
∑hexose	Hex	HMDB0000122
Energy metabolites		
α-ketobutyric acid	Ketobutyric acid	HMDB0000005
α-ketoglutaric acid	Ketoglutaric acid	HMDB0000208
cis-Aconitic acid	Aconitic acid	HMDB0000072
Cyclic AMP	cAMP	HMDB0000058
Dihydroxyacetonephosphate	DHAP	HMDB0001473
Fumaric acid	Fumaric acid	HMDB0000134
Glutathione (reduced)	GSH	HMDB0000125
Glutathione disulfide (oxidized)	GSSG	HMDB0003337
Glucose 6-phosphate	Hexose-phosphate	HMDB0001401
Lactic acid	Lactic acid	HMDB0000190
Oxaloacetic acid	Oxaloacetic acid	HMDB0000223
D-Ribulose 5-phosphate	Pentose-phosphate	HMDB0000618
Phosphoenolpyruvic acid	PEP	HMDB0000263
Pyruvic acid	Pyruvic acid	HMDB0000243
Succinic acid	Succinic acid	HMDB0000254
D-erythrose-4-phosphate	Tetrose-phosphate	HMDB0001321
Fatty acids		
Capric acid	FA C10:0	HMDB0000511
Myristic acid	FA C14:0	HMDB0000806
Palmitic acid	FA C16:0	HMDB0000220
Palmitoleic acid	FA C16:1ω7	HMDB0003229
Stearic acid	FA C18:0	HMDB0000827
Oleic acid	FA C18:1ω9	HMDB0000207
Linoleic acid	FA C18:2ω6	HMDB0000673
Linolenic acid	FA C18:3ω3	HMDB0001388
Eicosadienoic acid	FA C20:2ω6	HMDB0005060
Eicosatrienoic acid	FA C20:3ω3	HMDB0010378
Dihomo-γ-linolenic acid	FA C20:3ω6	HMDB0002925
Arachidonic acid	FA C20:4ω6	HMDB0001043
Eicosapentaenoic acid	FA C20:5ω3	HMDB0001999
Adrenic acid	FA C22:4ω6	HMDB0002226
all-cis-4,8,12,15,19-docosapentaenoic acid	FA C22:5ω3c1	HMDB0039133
all-cis-7,10,13,16,19-docosapentaenoic acid	FA C22:5ω3c2	HMDB0006528
all-cis-4,7,10,13,16-docosapentaenoic acid	FA C22:5ω6	HMDB0001976
Docosahexaenoic acid	FA C22:6ω3	HMDB0002183
Lysophosphatidylcholines		
Lysophosphatidylcholine acyl C14:0	lysoPC a C14:0	HMDB0010379
Lysophosphatidylcholine acyl C16:0	lysoPC a C16:0	HMDB0010382
Lysophosphatidylcholine acyl C16:1	lysoPC a C16:1	HMDB0010383
Lysophosphatidylcholine acyl C17:0	lysoPC a C17:0	HMDB0012108
Lysophosphatidylcholine acyl C18:0	lysoPC a C18:0	HMDB0010384
Lysophosphatidylcholine acyl C18:1	lysoPC a C18:1	HMDB0002815

Lysophosphatidylcholine acyl C18:2	lysoPC a C18:2	HMDB0010386
Lysophosphatidylcholine acyl C20:3	lysoPC a C20:3	HMDB0010393
Lysophosphatidylcholine acyl C20:4	lysoPC a C20:4	HMDB0010396
Lysophosphatidylcholine acyl C24:0	lysoPC a C24:0	HMDB0010405
Lysophosphatidylcholine acyl C26:1	lysoPC a C26:1	HMDB0029220
Lysophosphatidylcholine acyl C28:0	lysoPC a C28:0	HMDB0029206
Lysophosphatidylcholine acyl C28:1	lysoPC a C28:1	HMDB0029221
Phosphatidylcholines		
Phosphatidylcholine diacyl C24:0	PC aa C24:0	-
Phosphatidylcholine diacyl C26:0	PC aa C26:0	-
Phosphatidylcholine diacyl C28:1	PC aa C28:1	HMDB0007867
Phosphatidylcholine diacyl C30:0	PC aa C30:0	HMDB0007934
Phosphatidylcholine diacyl C30:2	PC aa C30:2	HMDB0007903
Phosphatidylcholine diacyl C32:0	PC aa C32:0	HMDB0000564
Phosphatidylcholine diacyl C32:1	PC aa C32:1	HMDB0007904
Phosphatidylcholine diacyl C32:2	PC aa C32:2	HMDB0007906
Phosphatidylcholine diacyl C32:3	PC aa C32:3	HMDB0008163
Phosphatidylcholine diacyl C34:1	PC aa C34:1	HMDB0007911
Phosphatidylcholine diacyl C34:2	PC aa C34:2	HMDB0008029
Phosphatidylcholine diacyl C34:3	PC aa C34:3	HMDB0008192
Phosphatidylcholine diacyl C34:4	PC aa C34:4	HMDB0008232
Phosphatidylcholine diacyl C36:0	PC aa C36:0	HMDB0007886
Phosphatidylcholine diacyl C36:1	PC aa C36:1	HMDB0007887
Phosphatidylcholine diacyl C36:2	PC aa C36:2	HMDB0008070
Phosphatidylcholine diacyl C36:3	PC aa C36:3	HMDB0007981
Phosphatidylcholine diacyl C36:4	PC aa C36:4	HMDB0007983
Phosphatidylcholine diacyl C36:5	PC aa C36:5	HMDB0007984
Phosphatidylcholine diacyl C36:6	PC aa C36:6	HMDB0008657
Phosphatidylcholine diacyl C38:0	PC aa C38:0	HMDB0007985
Phosphatidylcholine diacyl C38:1	PC aa C38:1	HMDB0008269
Phosphatidylcholine diacyl C38:3	PC aa C38:3	HMDB0008271
Phosphatidylcholine diacyl C38:4	PC aa C38:4	HMDB0008304
Phosphatidylcholine diacyl C38:5	PC aa C38:5	HMDB0008522
Phosphatidylcholine diacyl C38:6	PC aa C38:6	HMDB0008751
Phosphatidylcholine diacyl C40:1	PC aa C40:1	HMDB0008791
Phosphatidylcholine diacyl C40:2	PC aa C40:2	HMDB0008564
Phosphatidylcholine diacyl C40:3	PC aa C40:3	HMDB0008183
Phosphatidylcholine diacyl C40:4	PC aa C40:4	HMDB0008536
Phosphatidylcholine diacyl C40:5	PC aa C40:5	HMDB0008599
Phosphatidylcholine diacyl C40:6	PC aa C40:6	HMDB0008688
Phosphatidylcholine diacyl C42:0	PC aa C42:0	HMDB0008537
Phosphatidylcholine diacyl C42:1	PC aa C42:1	HMDB0008538
Phosphatidylcholine diacyl C42:2	PC aa C42:2	HMDB0008539
Phosphatidylcholine diacyl C42:4	PC aa C42:4	HMDB0008798
Phosphatidylcholine diacyl C42:5	PC aa C42:5	HMDB0008606
Phosphatidylcholine diacyl C42:6	PC aa C42:6	HMDB0008607
Phosphatidylcholine diacyl C48:0	PC aa C48:0	HMDB0008782
Phosphatidylcholine acyl-alkyl C30:0	PC ae C30:0	HMDB0013341
Phosphatidylcholine acyl-alkyl C30:1	PC ae C30:1	HMDB0013402
Phosphatidylcholine acyl-alkyl C30:2	PC ae C30:2	HMDB0013410

Phosphatidylcholine acyl-alkyl C32:1	PC ae C32:1	HMDB0013404
Phosphatidylcholine acyl-alkyl C32:2	PC ae C32:2	HMDB0013411
Phosphatidylcholine acyl-alkyl C34:0	PC ae C34:0	HMDB0013405
Phosphatidylcholine acyl-alkyl C34:1	PC ae C34:1	HMDB0013412
Phosphatidylcholine acyl-alkyl C34:2	PC ae C34:2	HMDB0011151
Phosphatidylcholine acyl-alkyl C34:3	PC ae C34:3	HMDB0013413
Phosphatidylcholine acyl-alkyl C36:0	PC ae C36:0	HMDB0013417
Phosphatidylcholine acyl-alkyl C36:1	PC ae C36:1	HMDB0013414
Phosphatidylcholine acyl-alkyl C36:2	PC ae C36:2	HMDB0013418
Phosphatidylcholine acyl-alkyl C36:3	PC ae C36:3	HMDB0013429
Phosphatidylcholine acyl-alkyl C36:4	PC ae C36:4	HMDB0013435
Phosphatidylcholine acyl-alkyl C36:5	PC ae C36:5	HMDB0013415
Phosphatidylcholine acyl-alkyl C38:0	PC ae C38:0	HMDB0013408
Phosphatidylcholine acyl-alkyl C38:1	PC ae C38:1	HMDB0013416
Phosphatidylcholine acyl-alkyl C38:2	PC ae C38:2	HMDB0013431
Phosphatidylcholine acyl-alkyl C38:3	PC ae C38:3	HMDB0013439
Phosphatidylcholine acyl-alkyl C38:5	PC ae C38:5	HMDB0013432
Phosphatidylcholine acyl-alkyl C38:6	PC ae C38:6	HMDB0013409
Phosphatidylcholine acyl-alkyl C40:1	PC ae C40:1	HMDB0013433
Phosphatidylcholine acyl-alkyl C40:2	PC ae C40:2	HMDB0013437
Phosphatidylcholine acyl-alkyl C40:3	PC ae C40:3	HMDB0013445
Phosphatidylcholine acyl-alkyl C40:4	PC ae C40:4	HMDB0013442
Phosphatidylcholine acyl-alkyl C40:5	PC ae C40:5	HMDB0013444
Phosphatidylcholine acyl-alkyl C40:6	PC ae C40:6	HMDB0013422
Phosphatidylcholine acyl-alkyl C42:0	PC ae C42:0	HMDB0013423
Phosphatidylcholine acyl-alkyl C42:1	PC ae C42:1	HMDB0013434
Phosphatidylcholine acyl-alkyl C42:2	PC ae C42:2	HMDB0013438
Phosphatidylcholine acyl-alkyl C42:3	PC ae C42:3	HMDB0013458
Phosphatidylcholine acyl-alkyl C42:4	PC ae C42:4	HMDB0013448
Phosphatidylcholine acyl-alkyl C42:5	PC ae C42:5	HMDB0013451
Phosphatidylcholine acyl-alkyl C44:3	PC ae C44:3	HMDB0013449
Phosphatidylcholine acyl-alkyl C44:4	PC ae C44:4	HMDB0013460
Phosphatidylcholine acyl-alkyl C44:5	PC ae C44:5	HMDB0013456
Phosphatidylcholine acyl-alkyl C44:6	PC ae C44:6	HMDB0013450
Sphingolipids		
Hydroxysphingomyelin C14:1	SM (OH) C14:1	HMDB0013462
Hydroxysphingomyelin C16:1	SM (OH) C16:1	HMDB0013463
Hydroxysphingomyelin C22:1	SM (OH) C22:1	HMDB0013466
Hydroxysphingomyelin C22:2	SM (OH) C22:2	HMDB0013467
Hydroxysphingomyelin C24:1	SM (OH) C24:1	HMDB0013469
Sphingomyelin C16:0	SM C16:0	HMDB0010169
Sphingomyelin C16:1	SM C16:1	HMDB0013464
Sphingomyelin C18:0	SM C18:0	HMDB0012087
Sphingomyelin C18:1	SM C18:1	HMDB0012101
Sphingomyelin C20:2	SM C20:2	HMDB0013465
Sphingomyelin C22:3	SM C22:3	HMDB0013468
Sphingomyelin C24:0	SM C24:0	HMDB0011697
Sphingomyelin C24:1	SM C24:1	HMDB0012107
Sphingomyelin C26:0	SM C26:0	HMDB0011698
Sphingomyelin C26:1	SM C26:1	HMDB0013461

Table S6. Percent recoveries of standard reference materials (SRMs) used for metabolomic analyses in blubber, liver and plasma of Eastern Beaufort Sea belugas and ringed seals from Lake Melville. The full name of each metabolite can be found in Table S5.

**Percent recovery of SRMs of belugas** (%)		**Percent recovery of SRMs of ringed seals** (%)	
Blubber			
Glu	142 %	PC aa C36:2	142 %
Ala	135 %	Succinic acid	129 %
Taurine	132 %	Sarcosine	122 %
Val	121 %	SM C16:0	118 %
Gly	117 %	SM C18:1	114 %
His	110 %	Val	103 %
Gln	101 %	Taurine	102 %
lysoPC a C18:2	100 %	FA C22:6	100 %
Carnosine	97 %	lysoPC a C18:2	99 %
GCDCA	95 %	AC C0	98 %
AC C0	89 %	TCDCA	96 %
TCDCA	89 %	GCDCA	96 %
PC ae C34:0	86 %	UDCA	94 %
SM C16:0	77 %	PC ae C36:2	92 %
Ac-Orn	55 %	lysoPC a C17:0	91 %
		Gln	89 %
		Glu	89 %
		Ala	87 %
		Pro	84 %
		AC C16:2	80 %
		Tetrose-phosphate	79 %
		FA C20:4	77 %
		Tyr	76 %
		FA C20:5	70 %
		Asn	65 %
Liver			
FA C20:5	149 %	Ac-Orn	138 %
FA C22:6	142 %	Succinic acid	126 %
FA C20:4	130 %	PC aa C36:2	126 %
Taurine	128 %	AC C0	120 %
lysoPC a C18:0	123 %	FA C18:1	117 %
Ala	107 %	FA C14:0	112 %
AC C0	105 %	Tetrose-phosphate	109 %
GCDCA	103 %	Phosphoenolpyruvate	104 %
Glu	103 %	Carnosine	101 %
TCDCA	101 %	Ala	96 %
SM C26:0	101 %	Glu	95 %
Gly	98 %	SM (OH) C24:1	93 %

His	96 %	lysoPC a C18:0	91 %
Gln	92 %	Gly	89 %
Met	91 %	GCDCA	89 %
Ser	89 %	TCDCA	86 %
Hexose-phosphate	83 %	His	84 %
PC aa C36:2	80 %	Pro	83 %
Ac-Orn	69 %	Gln	80 %
Succinic acid	65 %	Taurine	77 %
		Tyr	67 %
		FA C20:4	64 %
Plasma			
Ala	148 %	FA C20:4	119 %
Arg	148 %	Leu	110 %
Phe	144 %	PC aa C36:2	106 %
His	144 %	FA C22:6	106 %
Ser	144 %	Ile	103 %
PC aa C36:2	93 %	Lys	102 %
CDCA	91 %	PC ae C34:1	98 %
GCDCA	81 %	Ala	97 %
FA C22:5n6c	80 %	Arg	94 %
GDCA	78 %	PC aa C34:2	94 %
PC aa C34:2	74 %	Phe	93 %
PC ae C34:1	73 %	Succinic acid	92 %
Hex	72 %	His	90 %
Lactic acid	61 %	a-ketoglutaric acid	88 %
		GDCA	87 %
		FA C16:1	87 %
		Ser	85 %
		CDCA	80 %
		Hex	76 %
		GCDCA	66 %

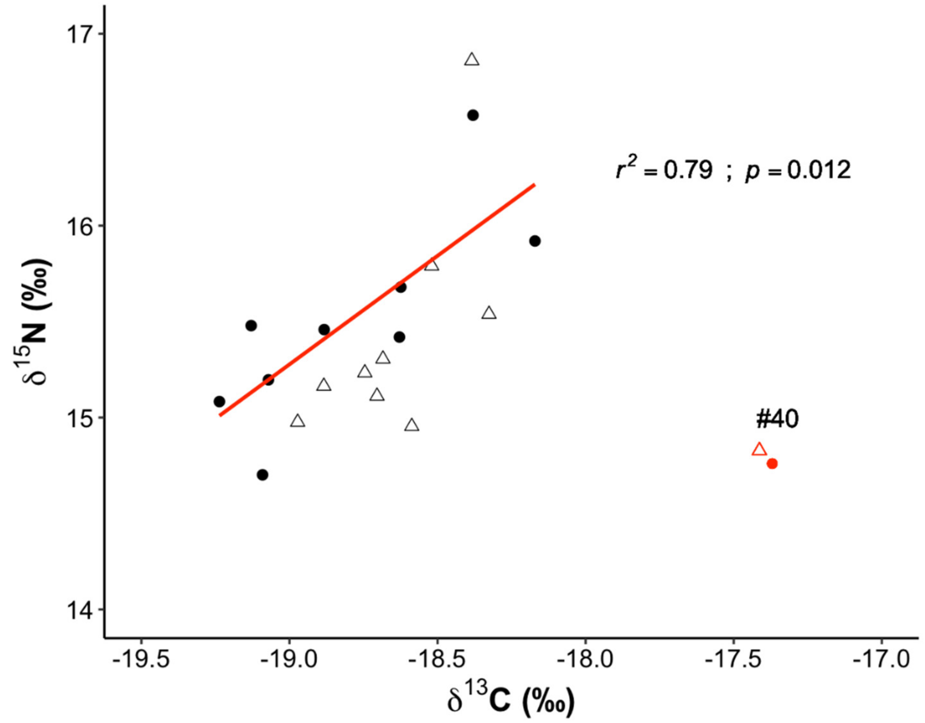

Figure S1. Correlation between muscle (●) and liver (△) δ^{15}N and δ^{13}C values (‰) in ringed seals of Lake Melville. Carbon and nitrogen stable isotope ratios were significantly correlated in muscle (red line), but not in liver ($p = 0.07$). Values of δ^{13}C have been determined from lipid-extracted samples. The ringed seal #40 has been added to the graph to better illustrate its outlierness. This individual has not been included in the correlation analysis between δ^{15}N and δ^{13}C values.

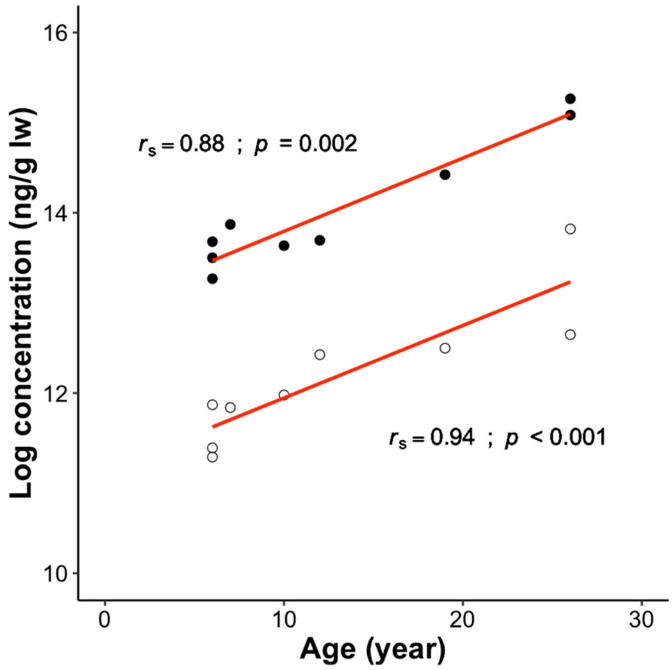

Figure S2. Correlation between age of seals and log-transformed Σ_{124}PCB (●) and Σ_{24}PBDE (○) blubber concentrations in ringed seal blubber ($n = 9$) from Lake Melville.

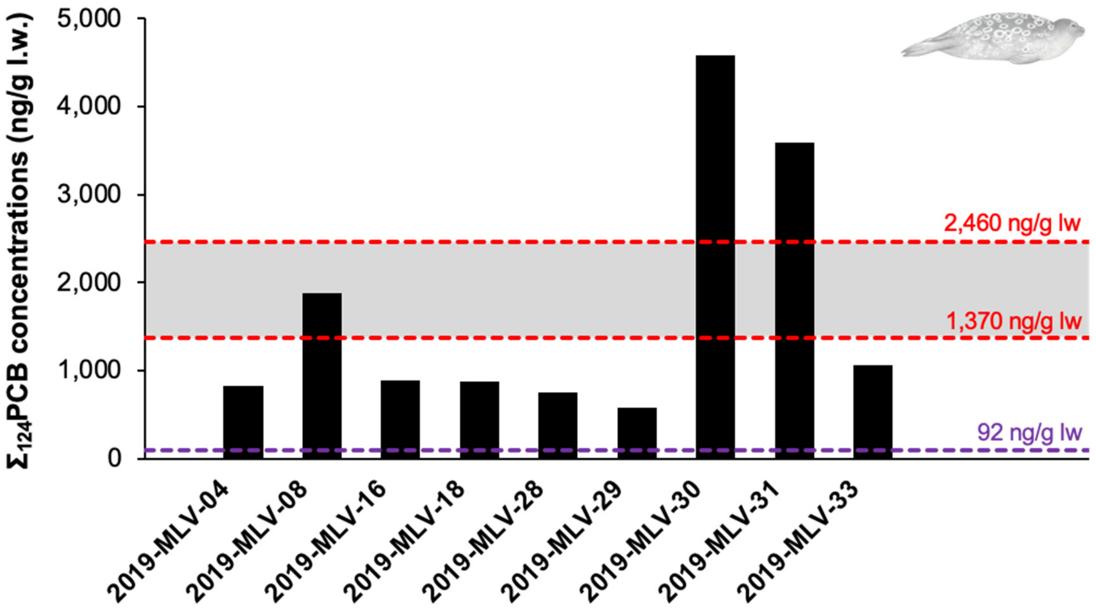

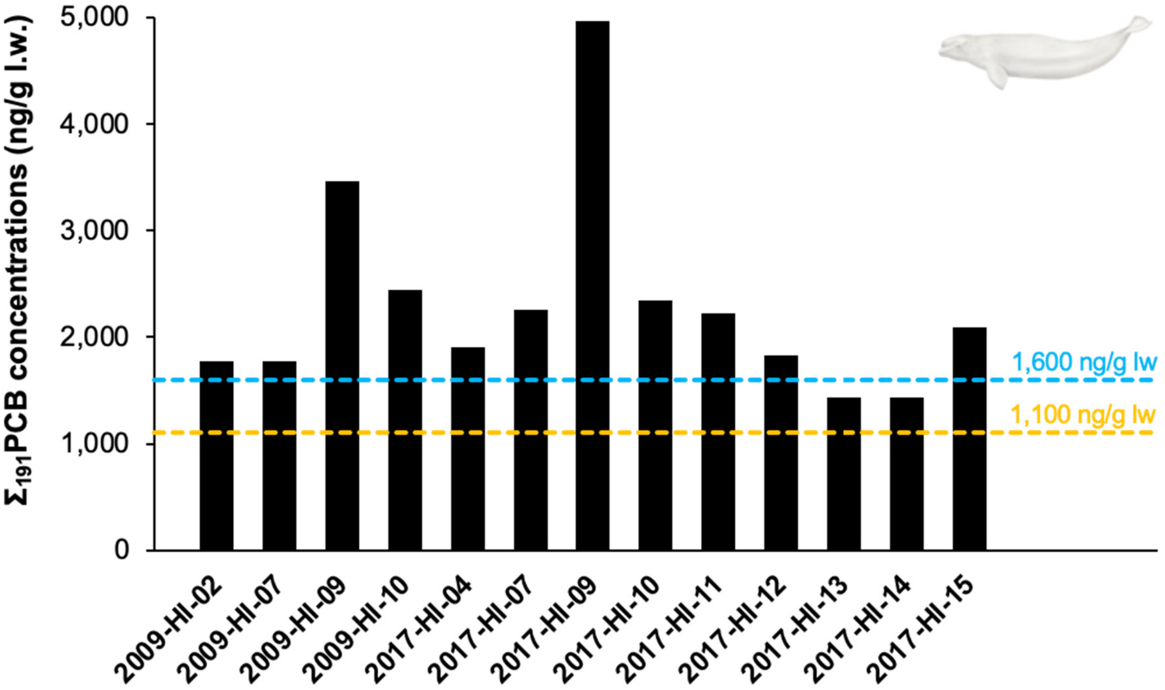

Figure S3. Blubber concentrations (ng/g lw) of ΣPCB in ringed seals (n = 9) from Lake Melville (top chart) and in belugas (n = 13) from Eastern Beaufort Sea (bottom chart). The grey band represents the range of PCB effect thresholds estimated by Brown et al. (2014) for five genes (*Ahr*, *Esr1*, *Igf1*, *Il1b*, and *Nr3c1*) in ringed seal liver. The dashed lines represent effect thresholds of PCBs for the disruption of vitamin A and E profiles in belugas (blue; Desforges et al., 2013), and for phagocytosis in cetaceans (yellow; Desforges et al., 2016), and for lymphocyte proliferation in ringed seals (purple; Desforges et al., 2016).

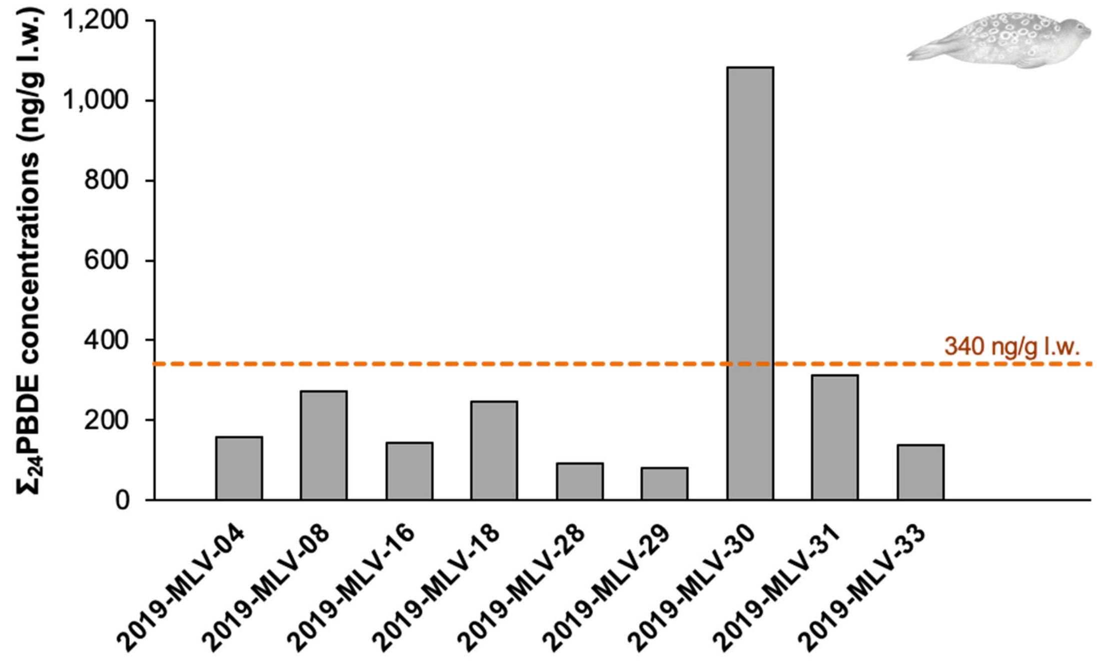

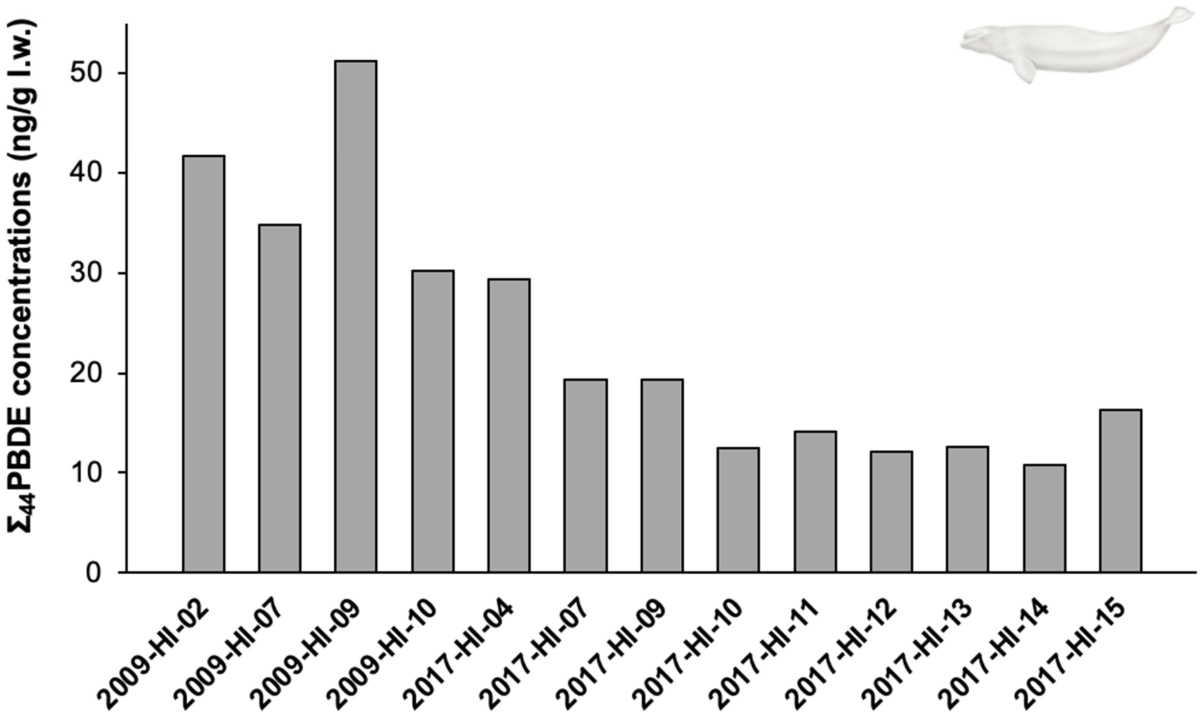

Figure S4. Blubber concentrations (ng/g lw) of ΣPBDE in ringed seals (n = 9) from Lake Melville (top chart) and in belugas (n = 13) from Eastern Beaufort Sea (bottom chart). The orange dashed line represent the effect threshold of PBDEs for phagocytosis in harbor seals (Desforges et al., 2016).

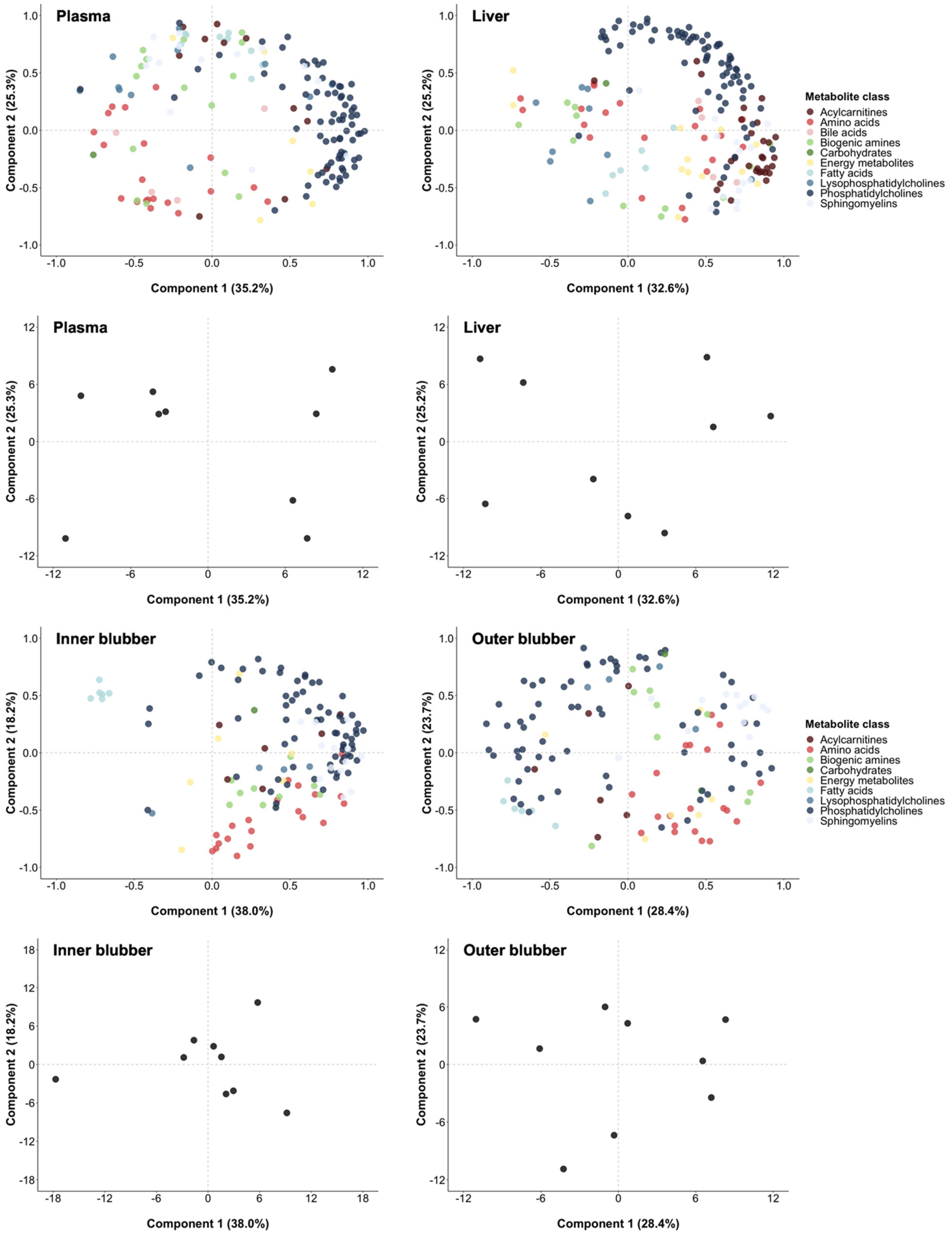

Figure S5. Score and loadings plots of PCAs performed with log-transformed percent contributions of metabolites quantified in plasma, liver, inner blubber and outer blubber of ringed seals from Lake Melville. Observations and variables are projected onto components 1 and 2 of PCAs.

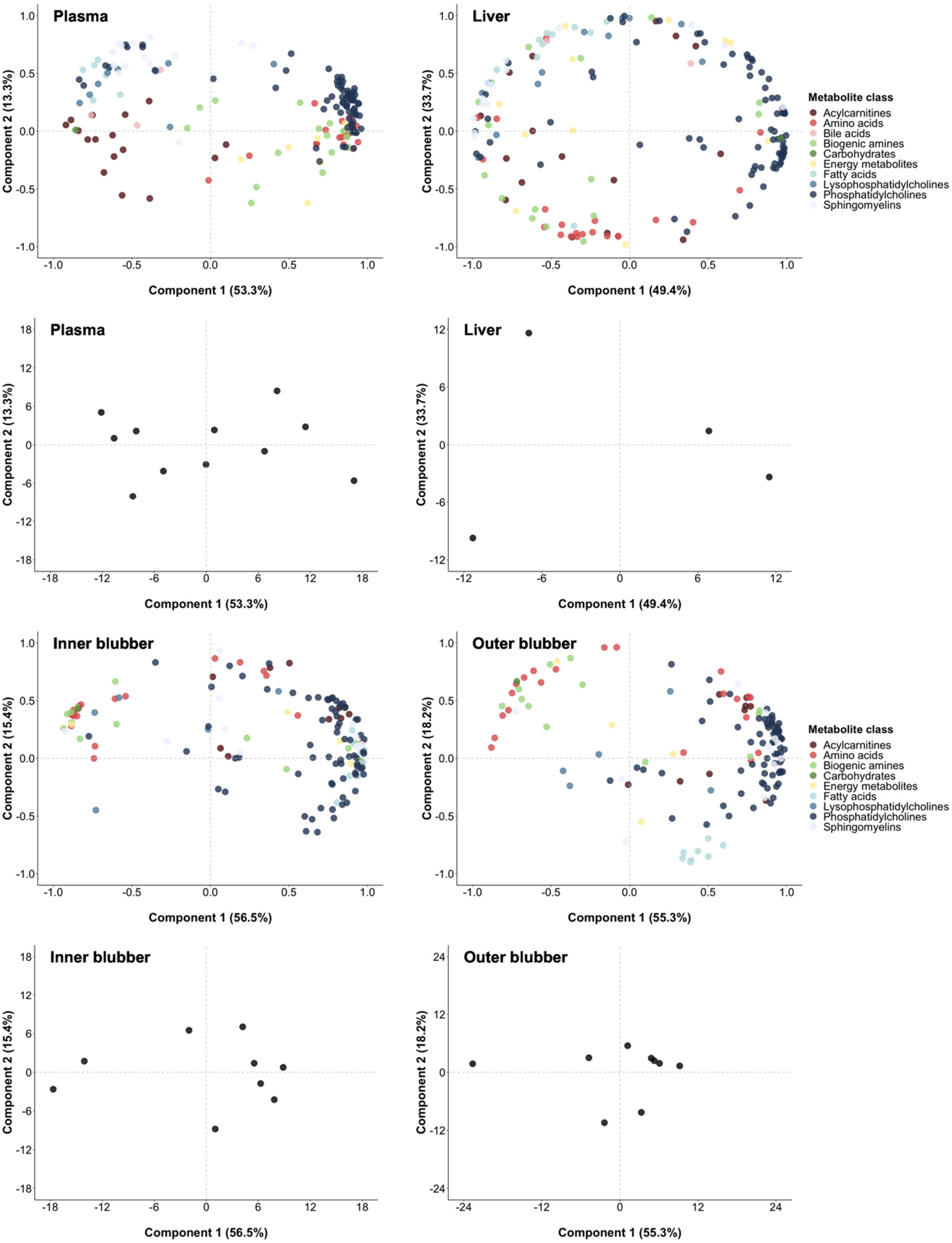

Figure S6. Score and loadings plots of PCAs performed with log-transformed percent contributions of metabolites quantified in plasma, liver, inner blubber and outer blubber of Eastern Beaufort Sea belugas. Observations and variables are projected onto components 1 and 2 of PCAs.

References

1. Brown, T.M., Ross, P.S., Reimer, K.J., Veldhoen, N., Dangerfield, N.J., Fisk, A.T., Helbing, C.C., 2014. PCB related effects thresholds as derived through gene transcript profiles in locally contaminated ringed seals (Pusa hispida). Environ. Sci. Technol. 48, 12952–12961. https://doi.org/10.1021/es5032294
2. Desforges, J.-P.W., Ross, P.S., Dangerfield, N., Palace, V.P., Whiticar, M., Loseto, L.L., 2013. Vitamin A and E profiles as biomarkers of PCB exposure in beluga whales (Delphinapterus leucas) from the western Canadian Arctic. Aquat. Toxicol. 142–143, 317–328. https://doi.org/http://dx.doi.org/10.1016/j.aquatox.2013.08.004
3. Desforges, J.-P.W., Sonne, C., Levin, M., Siebert, U., De Guise, S., Dietz, R., 2016. Immunotoxic effects of environmental pollutants in marine mammals. Environ. Int. 86, 126–139. https://doi.org/http://dx.doi.org/10.1016/j.envint.2015.10.007
